# Temporal transcriptomic profiling of channel catfish (*Ictalurus punctatus*) gills after acute copper exposure reveals suppressed immune gene responsiveness to *Flavobacterium covae* infection

**DOI:** 10.3389/fvets.2026.1855010

**Published:** 2026-07-16

**Authors:** Jason W. Abernathy, Yesutor K. Soku, Nithin Muliya Sankappa, Miles D. Lange, Bradley D. Farmer, Benjamin H. Beck, David L. Straus

**Affiliations:** 1 United States Department of Agriculture, Agricultural Research Service, Aquatic Animal Health Research Unit (AAHRU), Auburn, AL, United States; 2 Department of Pathobiology, College of Veterinary Medicine, Tuskegee University, Tuskegee, AL, United States; 3 Oak Ridge Institute for Science and Education (ORISE), ARS Research Participation Program, Oak Ridge, TN, United States; 4 United States Department of Agriculture, Agricultural Research Service, Harry K. Dupree Stuttgart National Aquaculture Research Center (HKDSNARC), Stuttgart, AR, United States

**Keywords:** channel catfish (*Ictalurus punctatus*), copper sulfate, *Flavobacterium covae*, host-pathogen, immunocompentence, RNA sequencing, stress response, transcriptome

## Abstract

Copper sulfate pentahydrate (CuSO_4_·5H_2_O) is widely used as a chemical prophylactic in aquaculture, yet its immunomodulatory effects in fish remain poorly understood. Here, we investigated how the timing of CuSO_4_ exposure shapes early immune responses and survival outcomes in channel catfish (*Ictalurus punctatus*) challenged with *Flavobacterium covae*. Channel catfish fingerlings were exposed to 2.1 mg/L CuSO_4_ either immediately before *F. covae* infection or following a 24 h recovery period, and gill transcriptomes were profiled at 1, 4, and 24 h post-challenge. Infection alone induced strong, time-structured innate and adaptive immune activation, including robust cytokine, chemokine, and pattern-recognition receptor (PRR) signaling. In contrast, Cu exposure followed by immediate infection produced no differentially expressed genes at 1 h and only a minimal response at 4 h, indicating a profound early suppression of host immune activation and a shift toward cellular stress-mitigation pathways. Fish that were treated with Cu followed by a 24 h recovery and then infected with *F. covae* regained immune responsiveness, displaying coordinated innate and adaptive activation by 4–24 h, consistent with significantly improved survival relative to immediate Cu exposure. Together, these results demonstrate that Cu immunological impact is strongly time-dependent. Acute exposure suppresses early immune readiness and increases susceptibility, whereas a recovery interval restores immune competence and enhances resistance to *F. covae*. These findings provide mechanistic support for incorporating recovery periods into CuSO_4_ treatment protocols to avoid unintended immunosuppression in aquaculture.

## 1 Introduction

Aquaculture is an important constituent of food production worldwide, and the demand for fish has increased as the per capita consumption across the world is continually increasing. The increasing demand for fish production in aquaculture has resulted in an intensification of the culture systems. Intensification often leads to increased disease outbreaks, many times resulting in high mortality and economic losses. One of the most important bacterial species affecting aquatic and fish populations are collectively known as columnaris-causing bacteria (1), including *Flavobacterium columnare, F. oreochromis, F. psychrophilum*, and *F. covae*, the predominant causative agent of columnaris disease in freshwater fish (2, 3). This pathogen, often ubiquitous in freshwater fish farms, selectively attacks gills, skin, and fins when fish are stressed, causing necrosis of tissues and, in severe cases, killing the fish (4, 5). Channel catfish, *Ictalurus punctatus*, is one of the most important species in U.S. aquaculture, and has high vulnerability to Columnaris disease, especially at the fingerling stage when fish are more susceptible to environmental factors and infectious agents (6). Effective new disease management strategies for catfish are always needed to make catfish aquaculture sustainable (7–9).

Copper sulfate pentahydrate (denoted as CuSO_4_·5H_2_O) is an inorganic, multiuse chemical that has been used commonly in aquaculture owing to its broad-spectrum antimicrobial properties. Conventionally, this chemical has been functionally used in aquaculture as an algaecide, parasiticide, and bactericide (10, 11). Early studies demonstrated that it was effective in controlling ectoparasites and protozoan diseases, which are diseases common in an intensive form of fish farming (12). In the 1980s, scientists began to conduct studies on the use of CuSO_4_ as a method to control bacterial pathogens, including *Aeromonas hydrophila* and *F. columnare*, both agents of significant economic losses in commercial aquaculture (8, 13). In recent years, there has been a renewed focus on CuSO_4_, attributed to its potential in alleviating bacterial infections, such as those induced by *F. columnare* (14). The attractiveness of copper sulfate can be attributed to its cost-effectiveness and its efficacy in managing microbial proliferation, alongside its straightforward application process. Nonetheless, the pathways through which CuSO_4_ influences fish health, whether beneficially or adversely, remain intricate and occasionally contradictory (7–9, 15–22).

In channel catfish, Farmer et al. (14) examined the application of CuSO_4_ as a pretreatment aimed at lessening their vulnerability to columnaris disease. The study identified that CuSO_4_ exposure followed by a 24 h recovery period considerably improved survival rates when challenged with *F. columnare* afterward. This result holds particular significance because, for the first time, it showed evidence suggesting that proper recovery from the initial CuSO_4_ exposure may help fish increase their resilience to bacterial infections (fish farmers call this “hardening” the fish). The research indicated that CuSO_4_ has the potential to enhance a nonspecific immune response in fish, subsequently contributing to the attenuation of bacterial colonization and the advancement of disease (14). Previous research conducted by MacFarlane et al. (13) demonstrated that exposure to CuSO_4_ heightened the resistance of striped bass (*Morone saxatilis*) against *Flexibacter columnare*.

Nevertheless, the application of CuSO_4_ for disease control can be regarded as challenging in several ways. While the studies have indeed revealed that CuSO_4_ reduces populations of pathogens, this chemical can sometimes have harmful side effects because of improper use. Early on, Hetrick et al. (23) were able to show that sublethal exposure to copper increased the susceptibility of rainbow trout (*Oncorhynchus mykiss*) to Infectious Hematopoietic Necrosis Virus (IHNV), suggesting that copper has an immunosuppressive action within a narrow optimal concentration. A similar study by Knittel (24) showed that steelhead trout subjected to CuSO_4_ were more susceptible to redmouth infection (*Yersinia ruckeri*), further complicating the dynamics of copper toxicity in fish. These findings emphasize that optimizing dose and timing is crucial when CuSO_4_ is used in aquaculture for bacterial infection treatment (16–19).

The antimicrobial effects of copper (Cu) are extensively documented in the literature. Copper is a very complex metal with two oxidation states. Copper primarily exhibits oxidation states of +1 (cuprous) and +2 (cupric), with +2 being the more common and stable state; however, under certain conditions, it can also exist in a less common +3 oxidation state. Cu^2+^ has been hypothesized to interfere with bacterial cell membranes and block a critical metabolic pathway, such as oxidative phosphorylation and replication of DNA. At the same time, ionic interactions may replace other metal ions from their natural binding sites in proteins, causing structural changes that impair bacterial function (25). Besides the inherent antimicrobial action, Cu has been shown to stimulate the immune response in fish. Deficient concentrations of Cu^2+^ can increase the activity of immune cells like macrophages and neutrophils, which play a critical role in the defense mechanisms of fish against infectious agents (26, 27).

Despite its antimicrobial properties, Cu can be an inducer of stress in fish, particularly at high concentrations and with prolonged exposure. Copper toxicity is influenced by several environmental factors, including water hardness, alkalinity, and temperature (10, 12). In soft water, where the buffering capacity is low, Cu can become more toxic, leading to gill damage, impaired respiration, and increased mortality (28). The dose-response relationship of Cu has a very narrow therapeutic-to-toxic ratio, meaning that dosage and duration should be optimized to attain the desired antimicrobial effect to cause the least harm to fishes.

Copper sulfate pentahydrate application has remained part of current aquaculture practices and integrated disease management methods. Previous studies have been conducted on its use in conjunction with other treatments, like vaccination or probiotics, to improve fish health while reducing the risk of Cu toxicity. An illustrative study by Rougier et al. (29) demonstrated that minimal amounts of Cu, when utilized in conjunction with probiotic therapies, conferred a protective effect against *Listeria monocytogenes* in Zebrafish (*Danio rerio*), indicating that Cu has the potential for synergistic application alongside various health management strategies.

Another focus area is using Cu to prevent the build-up of biofilm in aquaculture. Biofilm, which consist of bacterial colonies that adhere to surfaces, can serve as reservoirs for pathogens and contribute to the persistence of infections in fish farms (30). Some studies have demonstrated that Cu can inhibit biofilm formation, reducing bacterial outbreak risks (31, 32). Its capability for controlling both free-swimming planktonic bacteria and pathogens associated with biofilms makes Cu an invaluable tool in aquaculture, especially in recirculating aquaculture systems (RAS) where water quality and pathogen control are at a premium.

In addition, the application of Cu to limit the use of antibiotics in aquaculture has become increasingly popular. There is a growing interest in alternatives to antibiotics for disease control amidst growing concern about antimicrobial resistance. Used judiciously, CuSO_4_ provides a chemical option that does not contribute to the development of antimicrobial resistance. However, it requires careful management to prevent environmental contamination due to its potential to bioaccumulate in sediments and have lethal effects on non-target organisms (33). Guidelines that limit the quantity of CuSO_4_ applied in aquaculture to make its use environmentally compatible have thus been established (34).

The current study investigates how timing of CuSO_4_ exposure affects the susceptibility and gene responsiveness of channel catfish fingerlings to *F. covae* infection. We extend previous works by Farmer et al. (7, 14, 35), using time-course of exposure (CuSO_4_ and *F. covae*) and multiple recovery periods (CuSO_4_) in a full factorial design experiment that assesses both fish survival and global gene expression response. The functional effects of treatment with CuSO_4_ on columnaris disease were assessed by performing bulk RNA sequencing on gill samples from control and CuSO_4_-treated fish either immediately (more susceptible) or 24 h after (more resistant) CuSO_4_ treatment followed by a challenge with *F. covae*. We conducted transcriptomic analyses over the time-course of infection between groups with a focus on identifying functional differences in immune regulation and stress response. Thus, the current study delineates the molecular mechanisms of Cu-mediated susceptibility/protection through the analysis of gill tissues from CuSO_4_-exposed fish challenged with *F. covae*.

## 2 Materials and methods

### 2.1 Fish husbandry and maintenance

Channel catfish (*Ictalurus punctatus*) were spawned and reared at the Harry K. Dupree Stuttgart National Aquaculture Research Center and raised in flow-through freshwater tanks at the facility. Fish were acclimated to the laboratory environment for 2 weeks before the experiment. Water quality parameters, including temperature (26 °C−28 °C), pH (7.0–8.0), and dissolved oxygen levels (>5 mg/L), were monitored daily and maintained within optimal ranges for channel catfish throughout the study period. Fish were fed a commercial diet *ad libitum* during the acclimation period, but feeding was halted 24 h before and during the experimental treatments.

### 2.2 Experimental design

The concentration of CuSO_4_ used in this study was 2.1 mg/L, based on the total alkalinity divided by 100 (a typical farm treatment) as well as previous research indicating its effectiveness in modulating fish susceptibility to bacterial infections at this level (14). Channel catfish fingerlings (average 7.3 ± 1.5 g) were split among a total of six treatment or control groups, as follows:

**Group 1:** Fish were exposed to 2.1 mg/L CuSO_4_ and then immediately challenged with *F. covae*. Gill tissue from individual fish (*n* = 6) were sampled at 1, 4, and 24 h after bacterial challenge.

**Group 2:** Fish were exposed to 2.1 mg/L CuSO_4_ and held statically for 24 h. Then fish were challenged with *F. covae*. Gill tissue from individual fish (*n* = 6) were sampled at 1, 4, and 24 h after bacterial challenge.

**Group 3:** Fish were exposed to 2.1 mg/L CuSO_4_ and then gill tissue from individual fish (*n* = 6) were sampled.

**Group 4:** Fish were exposed to 2.1 mg/L CuSO_4_ and held statically for 24 h. Then gill tissue from individual fish (*n* = 6) were sampled.

**Group 5:** Gill tissue from individual fish (*n* = 6) were sampled immediately prior to either CuSO_4_ and/or *F. covae* challenge to serve as no-treatment (0 hour) controls.

**Group 6:** Fish were challenged with *F. covae*. Gill tissue from individual fish (*n* = 6) were sampled at 1, 4, and 24 h after bacterial challenge.

### 2.3 Copper sulfate exposure

The CuSO_4_ solution was prepared by dissolving copper sulfate pentahydrate (CuSO_4_·5H_2_O) in double-distilled water to achieve the desired concentration. For the 24 h group, CuSO_4_ treatment was static then flow-through freshwater was turned on to tanks and fish allowed to recover for 24 h before either sampling or bacterial challenge. Each treatment group consisted of three replicate tanks, each containing 30 fish. Sampling of gill tissues for RNA extraction was performed at specific time points based on the experimental design. The exact groups are provided in Table 1.

**Table 1:** Description of the differentially expressed gene (DEG) comparison given the experimental design.

Name	Description
True control	Gill tissue from individual fish (*n* = 6) were sampled at 0 hours immediately prior to either copper sulfate and/or *F. covae* challenge to serve as no-treatment controls (**Group 5**).
*F. covae* 1h	Catfish were challenged with *F. covae*. Gill tissue from individual fish (*n* = 6) was sampled at 1 h after bacterial challenge (**Group 6**) and compared to **Group 5**. The comparison was named as *F. covae* 1h for future reference.
*F. covae* 4h	Catfish were challenged with *F. covae*. Gill tissue from individual fish (*n* = 6) was sampled at 4 h after bacterial challenge (**Group 6**) and compared to **Group 5**. The comparison was named *F. covae* 4 h for future reference.
*F. covae* 24h	Catfish were challenged with *F. covae*. Gill tissue from individual fish (*n* = 6) was sampled at 24 h after bacterial challenge (**Group 6**) and compared to **Group 5**. The comparison was named as *F. covae* 24 h for future reference.
*F. covae* Cu0h-1h	Catfish were exposed to 2.1 mg/L copper sulfate and then immediately challenged with *F. covae*. Gill tissue from individual fish (*n* = 6) was sampled at 1 h after bacterial challenge (**Group 1**) were compared with **Group 3**. The comparison was named *F. cov* Cu0h-1h for future reference.
*F. covae* Cu0h-4h	Catfish were exposed to 2.1 mg/L copper sulfate and then immediately challenged with *F. covae*. Gill tissue from individual fish (*n* = 6) was sampled at 4 h after bacterial challenge (**Group 1**) were compared with **Group 3**. The comparison was named *F. cov* Cu0h-4h for future reference.
*F. covae* Cu0h-24h	Catfish were exposed to 2.1 mg/L copper sulfate and then immediately challenged with *F. covae*. Gill tissue from individual fish (*n* = 6) was sampled at 24 h after bacterial challenge (**Group 1**) were compared with **Group 3**. The comparison was named *F. cov* Cu0h-24h for future reference.
Cu24h vs. Cu0h	Catfish were exposed to 2.1 mg/L copper sulfate and held statically for 24 h (**Group 4**) was compared with catfish exposed to 2.1 mg/L copper sulfate and then gill tissue from individual fish (*n* = 6) were sampled immediately (**Group 3**). The comparison was named Cu24h vs. Cu0h for future reference.
*F. covae* Cu24h-1h	Catfish were exposed to 2.1 mg/L copper sulfate and held statically for 24 h. Then fish were challenged with *F. covae*. Gill tissue from individual fish (*n* = 6) was sampled at 1 h after bacterial challenge (**Group 2**) and compared with **Group 4**. The comparison was named as *F. cov* Cu24h-1h for future reference.
*F. covae* Cu24h-4h	Catfish were exposed to 2.1 mg/L copper sulfate and held statically for 24 h. Then fish were challenged with *F. covae*. Gill tissue from individual fish (*n* = 6) was sampled at 4 h after bacterial challenge (**Group 2**) and compared with **Group 4**. The comparison was named as *F. cov* Cu24h-4h for future reference.
*F. covae* Cu24h-24h	Catfish were exposed to 2.1 mg/L copper sulfate and held statically for 24 h. Then fish were challenged with *F. covae*. Gill tissue from individual fish (*n* = 6) was sampled at 24 h after bacterial challenge (**Group 2**) and compared with **Group 4**. The comparison was named *F. cov* Cu24h-24h for future reference.

### 2.4 Bacterial challenge

*Flavobacterium covae* isolate LV-359-01 was retrieved from a frozen glycerol stock stored at −80 °C and streaked onto *F. covae* growth medium (FCGM) containing agar. After 48 h, bacterial colonies were dislodged from the agar using a sterile cotton swab and inoculated into 5 mL of FCGM medium. Planktonic suspensions were incubated at 28 °C for 24 h and then used to inoculate 1 L of FCGM that was then incubated for another 24 h in an orbital shaker at 200 rpm. After reaching an absorbance of 0.65 at 550 nm, the flask was removed and placed on a stir plate at room temperature. A 10 mL sample was removed for serial dilution and colony forming unit (CFU) enumeration (4.0 × 10^10^ bacteria/mL). For the challenges, 50 mL of bacterial suspension was added to 10 L tanks (2.0 × 10^8^ CFU/mL). After the addition of bacteria to tanks, the flow rate was set at 30 mL/min.

### 2.5 Survival curve analyses

Replicate tanks (*n* = 3) were also set up for survival curve analyses. Survival data were analyzed with SigmaPlot 11 (San Jose, CA) using Kaplan Meier Log Rank Survival Analysis and all pair-wise multiple comparisons used the Holm-Sidak method with adjusted *p* values. Treatment effects were considered significant at *p* < 0.05.

### 2.6 RNA extraction and sequencing

Gill samples were collected per the experimental design, with six individuals per treatment group representative of biological replicates. Gill samples were collected aseptically after euthanasia by an overdose (300 mg/L) of buffered MS-222 and immediately placed in an RNA stabilization solution (RNA*later*, ThermoFisher Scientific, Waltham, MA). The gill tissue samples were stored at −80 °C until needed. Total RNA was extracted using the Qiagen RNeasy Mini Kit (Germantown, MD) per the manufacturer's recommendation. Total RNA was treated with Amplification Grade DNase I (Sigma-Aldrich, Burlington, MA) and ethanol-precipitated prior to library construction. RNA integrity was assessed using an Agilent BioAnalyzer (Santa Clara, CA) and quantified using a NanoDrop spectrophotometer (Wilmington, DE). Total RNA was then standardized to 100 ng each sample and sequencing libraries were prepared using the NEBNext Ultra II Directional RNA Library Prep Kit for Illumina with the NEBNext Poly(A) mRNA Magnetic Isolation Module (New England Biolabs, Ipswich, MA). Barcodes used were the NEBNext Multiplex Oligos for Illumina (Index Primers Sets 1 and 2). Libraries were quantified using the NEBNext Library Quant Kit for Illumina, equimolar amounts pooled and sent to a service provider for sequencing (Novogene, Sacramento, CA). Libraries were sequenced on an Illumina HiSeq X Ten according to the manufacturer's instructions using a 2 × 150 bp paired end (PE) configuration.

### 2.7 Transcriptome analysis for the channel catfish gill tissue samples

Raw, demultiplexed reads at a minimum of 25 M PE reads/sample were delivered by the service provider. Bioinformatics was performed in house within the OmicsBox software (36) and R-bioconductor packages. Raw FASTQ files were initially preprocessed for quality control (QC) using FASTQC (37) and Trimmomatic (38) with the default parameters. This process removed low-quality bases, filtered out short reads, and eliminated any contaminating Illumina sequencing adapters. The channel catfish, *Ictalurus punctatus*, genome was obtained from the NCBI (assembly Coco_2.0; accession #GCA_001660625.3) and QC reads were aligned to it using the STAR aligner (39), with options of 2-pass mapping and overhang length set to 149. QC of the resultant BAM files was performed using RSeQC modules (40–42) to assess alignment quality and obtain quality scores, including Transcript Integrity Numbers (TIN). QC BAM files were then used to generate a gene-level counts data matrix for all samples via HTSeq software (43). Using HTSeq-count, the quantification level was set to feature type “exon” and grouped by “parent” attribute, strand specificity was set to “strand specific reverse,” and overlap mode was set to “union.” The R package edgeR (44) was then used to generate differentially expressed genes (DEGs). First, genes with low counts were filtered via the “filterByExpr” function followed by Weighted Trimmed mean of *M*-values (TMM) normalization. Generalized Linear Model (GLM) Quasi Likelihood *F*-Test was chosen for statistical assessment. A false discovery rate (FDR) adjusted *p*-value < 0.05 was set as the threshold for transcripts to be considered significant differentially expressed genes (DEGs) and transcripts were considered up- or downregulated if the log_2_ fold-change values were greater than one (logFC > 1) or less than negative one (logFC < – 1).

### 2.8 RNA sequencing pairwise comparisons

While there are many pairwise comparison combinations available per our experimental conditions (Table 1), we focused on the following per our interest in discovering functional differences among specific treatment groups:

1. Time-course infection of *F. covae*

Catfish were challenged with *F. covae*. Gill tissue from individual fish (*n* = 6) were sampled at 1, 4, and 24 h after bacterial challenge (**Group 6**) and compared to 0-hour control (**Group 5)**. The comparisons were named *F. covae* 1, 4, and 24 h for reference.

2. Treatment effect of acute copper sulfate on *F. covae* infection (more susceptible)

Catfish were exposed to 2.1 mg/L copper sulfate and then immediately challenged with *F. covae*. Gill tissue from individual fish (*n* = 6) were sampled at 1, 4, and 24 h after bacterial challenge (**Group 1**) and compared with **Group 3**. The comparisons were named *F. cov* Cu0h-1h, *F. cov* Cu0h-4h, and *F. cov* Cu0h-24h for reference.

3. Treatment effect of recovery copper sulfate on *F. covae* infection (more resistant)

Catfish were exposed to 2.1 mg/L copper sulfate and held statically for 24 h. Then fish were challenged with *F. covae*. Gill tissue from individual fish (*n* = 6) sampled at 1, 4, and 24 h after bacterial challenge (**Group 2**) were compared with **Group 4**. The comparisons were named *F. cov* Cu24h-1h, *F. cov* Cu24h-4h, and *F. cov* Cu24h-24h for reference.

4. Treatment effect of copper sulfate—acute exposure compared to recovery

Gill tissues (*n* = 6) from catfish that were exposed to 2.1 mg/L copper sulfate and held statically for 24 h (**Group 4**) were compared with gill tissues (*n* = 6) from catfish exposed to 2.1 mg/L copper sulfate and then sampled immediately (**Group 3**). The comparison was named Cu24h vs. Cu0h for reference.

### 2.9 Validation of RNA-Seq by quantitative polymerase chain reaction (qPCR)

Based on the DEGs from different treatment groups, five specific primers (Table 2) were utilized to independently evaluate RNA-seq results via reverse transcription quantitative PCR (RT-qPCR). Each total RNA sample was assessed using spectrophotometry (Bio-Tek Cytation 1, Agilent Technologies, Palo Alto, CA) and Agilent TapeStation 4200 with RNA Integrity Numbers (RIN) > 8 before reverse transcription. Then, cDNA synthesis was performed using the LunaScript RT SuperMix Kit (New England Biolabs, Ipswich, MA). Reactions contained 4.0 μL of LunaScript RT SuperMix (5X) and template RNA (300 ng), and the volume was adjusted using nuclease-free water to 20 μL. As a control, to rule out the presence of DNA in the extracted sample, no-RT reactions were prepared for each of the samples along with no template controls (negative control). Reaction conditions for cDNA synthesis included primer annealing at 25 °C for 2 min, cDNA synthesis at 55 °C for 10 min, and heat inactivation at 95 °C for 1 min. cDNA was kept at−20 °C after transcription until RT-qPCR.

**Table 2:** Primers for selected genes used in RT-qPCR reactions for validation of RNA-Seq data.

Gene	Forward primer	Reverse primer	Product size (bp)	Reference
*il-1β*	AGCAGCAATCCAGTCACCTC	TCGGGAGCTGAGATATACTTAAACA	160	This study
*mmp9*	CGTGGTAACGAGGAACGGAA	GCGGTCCCAATACATCCTGT	163	This study
*aprt*	AGAGACCGGGAGTAGGTATGAA	AAACTGGAGTGTGTGAGCTGT	147	This study
*blec*	CCCTGTAAACCCGTGTGGTC	CACACCACAGCCCCATCATT	113	This study
*irg1*	TCTCCCGTGTCCGAGTAGAG	CTCTCATCACTCAGCGGCTT	154	This study
*18s*	GAGAAACGGCTACCACATCC	GATACGCTCATTCCGATTACAG	128	(45)

Using a LightCycler 480 System (Roche Diagnostics, Indianapolis, IN), RT-qPCR was performed to assess the expression levels of each gene in gill tissues. Reaction and cycling conditions were prepared following the manufacturer's instructions (NEB), with modifications. The Luna Universal qPCR Master Mix was used for RT-qPCR in 10 μL reactions. Each reaction included 5 μL of Luna Universal qPCR Master Mix (2X), 0.5 μL of forward primer (1 μM), 0.5 μL of reverse primer (1 μM), 2 μL of nuclease-free water, and 2 μL of cDNA diluted 1:10 with nuclease free water. Reactions were carried out in triplicate under the following conditions: 95 °C for 15 s, followed by 45 cycles at 95 °C for 15 s and 60 °C for 30 s, followed by melting curve analysis. Samples were run in parallel with the 18S rRNA gene as reference for normalization (45).

Relative gene expression was calculated using the 2^−ΔΔ*CT*^ method (46, 47), normalizing target gene Ct values to the geometric mean of the selected reference genes and expressing changes relative to the control group. Data were evaluated by analysis of variance (ANOVA) to determine differences in means among different groups and then further analyzed *post hoc* using Tukey's multiple comparison test. Differences in means among the groups were considered statistically significant when *p* < 0.05.

### 2.10 Functional characterization of gene expression data

Gene ontology (GO) analysis was performed to determine the biological function of DEGs. The GO analysis assigns biological processes, cellular components, and molecular functions terms using both the Fisher's Exact test against the channel catfish annotated transcriptome and Gene Set Enrichment Analyses (GSEA), where the default parameters were selected within the OmicsBox software. Significance was set at an FDR < 0.05.

## 3 Results

### 3.1 Survival analysis of channel catfish

**Figure 1:**
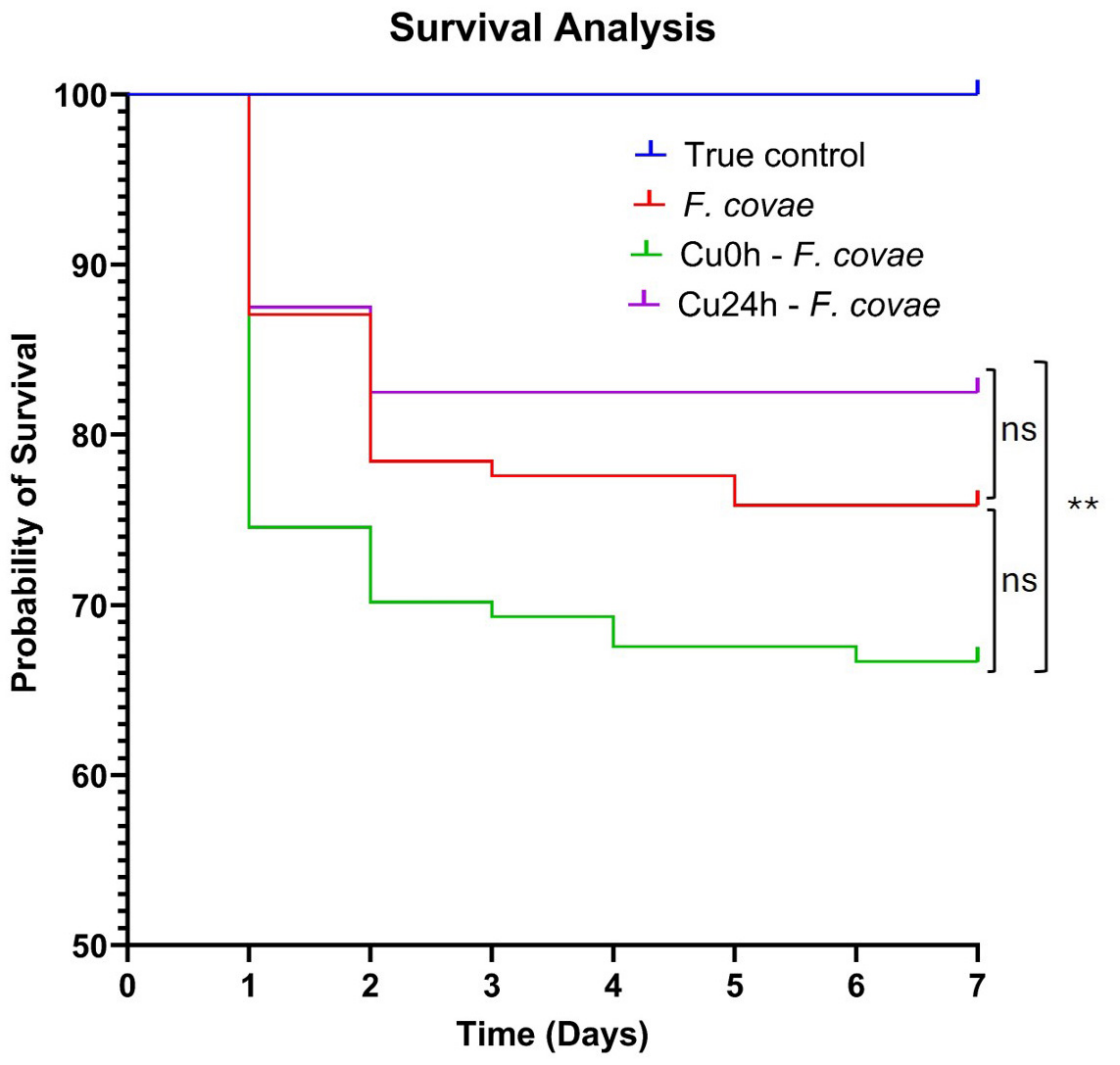
Kaplan–Meier survival curve of Channel Catfish exposed to CuSO_4_ for 0 and 24 h under static conditions and then challenged with *Flavobacterium covae*. Treatments were as follows: (1) fish exposed to 2.1 mg/L CuSO_4_ and then immediately challenged with *F. covae* (Cu0h- *F. covae*), (2) fish exposed to 2.1 mg/L CuSO_4_ and allowed recovery for 24 h then challenged with *F. covae* (Cu24h- *F. covae*), (3) fish not exposed to CuSO_4_ but challenged with *F. covae* (*F. covae*). **p < 0.05.

The survival analysis depicted in Figure 1 shows the probability of survival for three treatment groups of channel catfish fingerlings challenged with *Flavobacterium covae*. The survival probability for fish challenged with *F. covae* without any CuSO_4_ exposure decreased steadily over time. The final survival probability was approximately 76% by day 7. This indicates a moderate susceptibility of the fish to *F. covae* when no CuSO_4_ treatment was applied. Fish exposed to 2.1 mg/L CuSO_4_ and immediately challenged with *F. covae* showed a marked decrease in survival compared to the control group. Survival dropped sharply in the initial days and, by day 7, the survival probability was approximately 67%. This demonstrates that immediate CuSO_4_ exposure, without a recovery period, increased the fish's susceptibility to the bacteria, as previously demonstrated (14). The group exposed to 2.1 mg/L CuSO_4_ followed by a 24 h recovery period exhibited the highest survival among the treatment groups. The survival probability remained steady, with minimal mortality observed by day 7. The final survival percentage was approximately 83%. This shows that allowing a recovery period after CuSO_4_ exposure significantly enhances channel catfish resistance to *F. covae*, reducing mortality. Conversely, immediate exposure to the pathogen after CuSO_4_ treatment reduces survival, likely due to the stress and gill damage caused by copper introduction (14).

### 3.2 Differentially expressed genes (DEGs)

The RNA-seq analysis focused on comparisons among CuSO_4_-exposed groups at 0 h and after 24 h, and fish challenged with *F. covae* and sampled at 1, 4, and 24 h post-exposure. A breakdown of the significant findings on total DEGs showing upregulation or downregulation is provided in Table 3.

**Table 3:** Number of differentially expressed genes (DEGs) from each pairwise comparison.

Comparison	Upregulated genes	Downregulated genes	Total differentially expressed genes
*F. covae* 1h	868	544	1,412
*F. covae* 4h	2,097	2,747	4,844
*F. covae* 24h	1,494	1,571	3,065
*F. covae* Cu0h-1h	0	0	0
*F. covae* Cu0h-4h	32	13	45
*F. covae* Cu0h-24h	320	598	918
Cu24h vs. Cu0h	136	573	709
*F. covae* Cu24h-1h	42	89	131
*F. covae* Cu24h-4h	354	363	717
*F. covae* Cu24h-24h	1,553	2,372	3,925

#### 3.2.1 *F. covae*-only challenge

**Figure 2:**
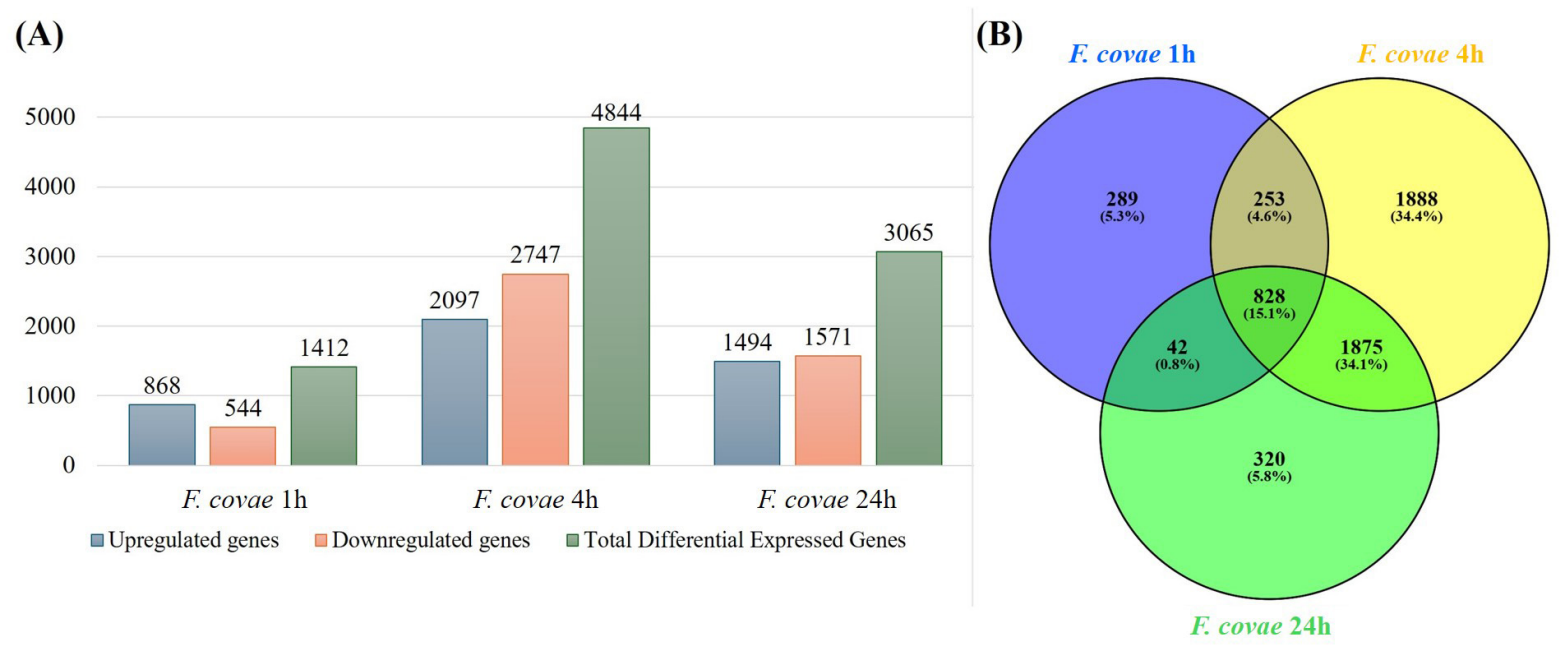
Differentially expressed genes (DEGs) from RNAseq analysis of channel catfish gill tissues after challenge with *F. covae*. Tissues were collected at 1 h, 4 h and 24 h after *F. covae* infection. **(A)** Bar diagram showing the upregulated, downregulated and total DEGs at each timepoint. **(B)** Venn diagram showing common DEGs at each timepoint.

In gill tissues of infected fish at 1 h post-infection, 1,412 DEGs were observed, with 868 genes upregulated and 544 genes downregulated (Figure 2A and Supplementary Table 1). This indicates an early and robust immune response, as expected during the initial stages of bacterial infection. At 4 h post-infection, the number of DEGs drastically increased to 4,844, with 2,097 upregulated and 2,747 downregulated genes reflecting a systematic inflammatory and stress response. At 24 h post-infection, 3,065 DEGs were identified, with 1,494 upregulated and 1,571 downregulated genes, consistent with a transition toward coordinated innate and adaptive immunity. A total of 828 common DEGs were found in *F. covae* 1, 4, and 24 h (Figure 2B), representing core responses to *F. covae*.

#### 3.2.2 CuSO_4_ exposure and immediate challenge with *F. covae*

**Figure 3:**
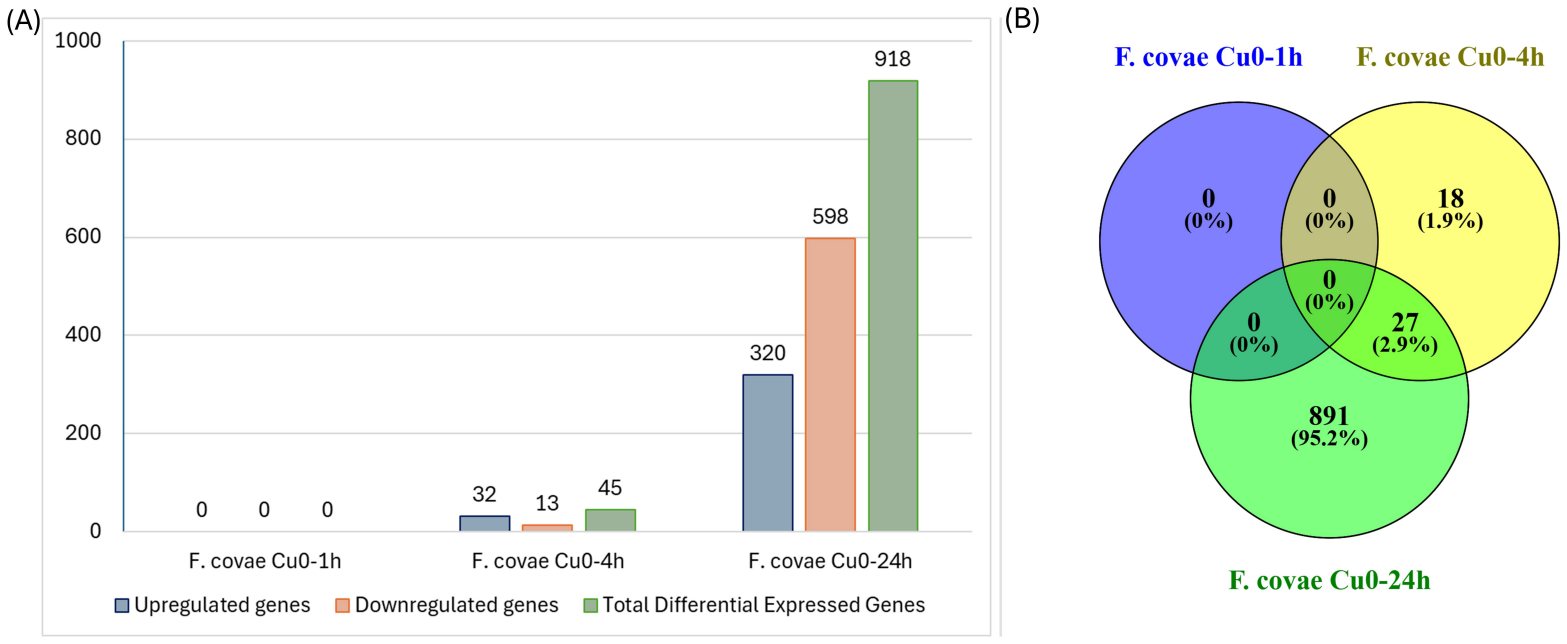
Differentially expressed genes (DEGs) from RNAseq analysis of channel catfish gill tissues exposed to copper at 2.1 mg/L and then challenged immediately with *F. covae*. Tissues were collected at 1 h, 4 h and 24 h after *F. covae* infection. **(A)** Bar diagram showing the upregulated, downregulated and total DEGs at each timepoint. **(B)** Venn diagram showing common DEGs at each timepoint.

Interestingly, CuSO_4_ exposure followed by immediate *F. covae* challenge showed minimal transcriptional response to infection early (0 DEGs at 1 h) while gradually increasing at 4 h (45 DEGs) up to 918 DEGs at the sampling endpoint of 24 h (Table 3; Figure 3A and Supplementary Table 2). These data indicate that acute CuSO_4_ exposure profoundly delays the canonical transcriptomic response to *F. covae*. Since no DEGs were detected at 1 h, no genes were shared across all three acute CuSO_4_ plus *F. covae* time points (Figure 3B). This pattern aligns with the reduced survival observed in this treatment group and is consistent with known copper-mediated inhibition of pattern recognition receptor (PRR) signaling and early immune activation (Discussed below).

#### 3.2.3 Effects of CuSO_4_ exposure alone (0 h vs. 24 h recovery)

Comparison of CuSO_4_-exposed uninfected fish sampled immediately vs. after 24 h revealed 709 DEGs (136 upregulated; 573 downregulated). This indicates that copper exposure induces strong stress-related transcriptional changes that partially resolve during recovery. Genes involved in metal detoxification, protein folding, and oxidative stress were enriched at acute exposure (0 h), whereas recovery was characterized by downregulation of these stress-associated pathways (Supplementary Table 3).

#### 3.2.4 CuSO_4_ exposure with 24 h recovery followed by *F. covae* challenge

**Figure 4:**
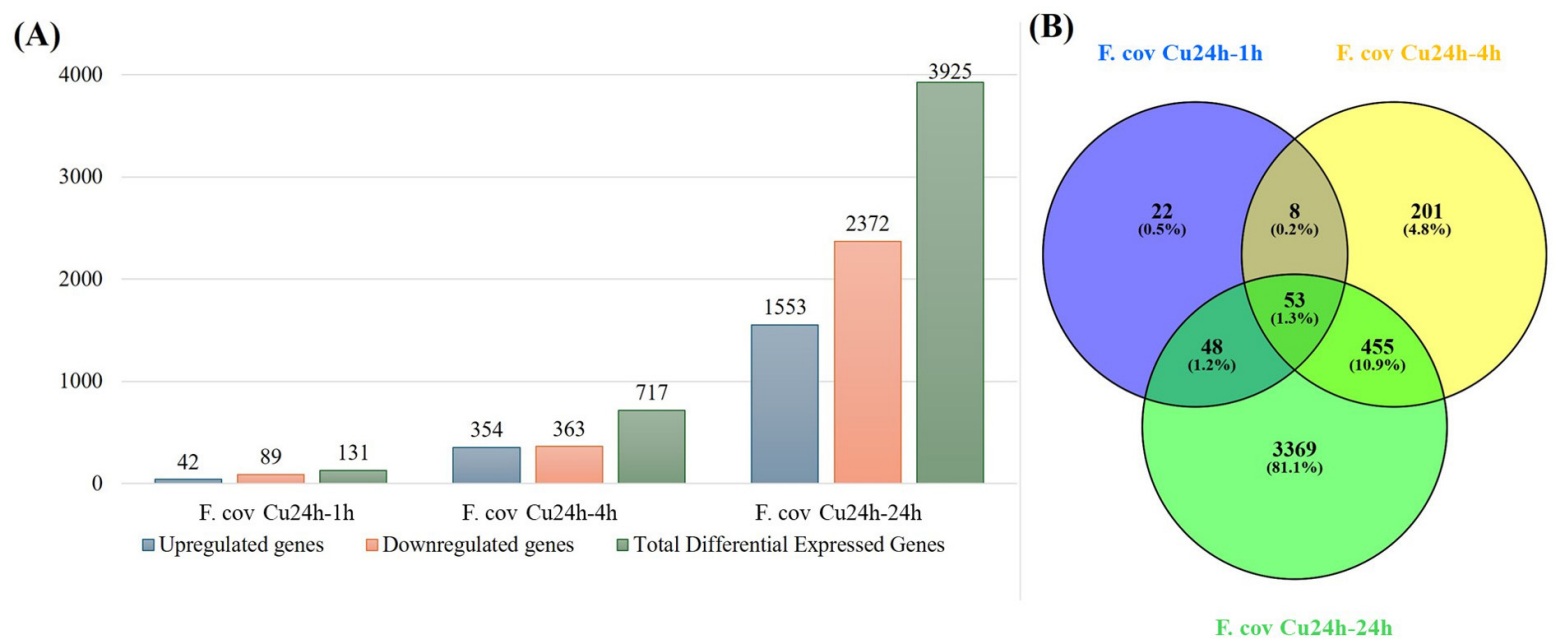
Differentially expressed genes (DEGs) from RNAseq analysis of channel catfish gill tissues exposed to copper at 2.1 mg/L and then challenged with *F. covae* infection after 24 h recovery period. Tissues were collected at 1 h, 4 h and 24 h after *F. covae* infection. **(A)** Bar diagram showing the upregulated, downregulated and total DEGs at each timepoint. **(B)** Venn diagram showing common DEGs at each timepoint.

In the early stages of infection (1 h), the number of DEGs was relatively small, with only 131 genes showing differential expression with 42 upregulated, and 89 downregulated (Figure 4A and Supplementary Table 4). The response at 4 h showed 717 DEGs, with a more even distribution of up- and downregulated genes (354 upregulated, 363 downregulated), suggesting a more gradual activation of immune pathways and early cellular responses to infection. The most substantial response was observed at the 24 h time-point. A total of 3,925 DEGs were detected, with 1,553 genes upregulated and 2,372 downregulated (Figure 4A and Supplementary Table 4). A total of 53 DEGs were shared across all 3 time-points (Figure 4B).

### 3.3 Gene ontology based on GSEA and Fisher's exact test

#### 3.3.1 Gene ontology *F. covae*-only challenge

**Figure 5:**
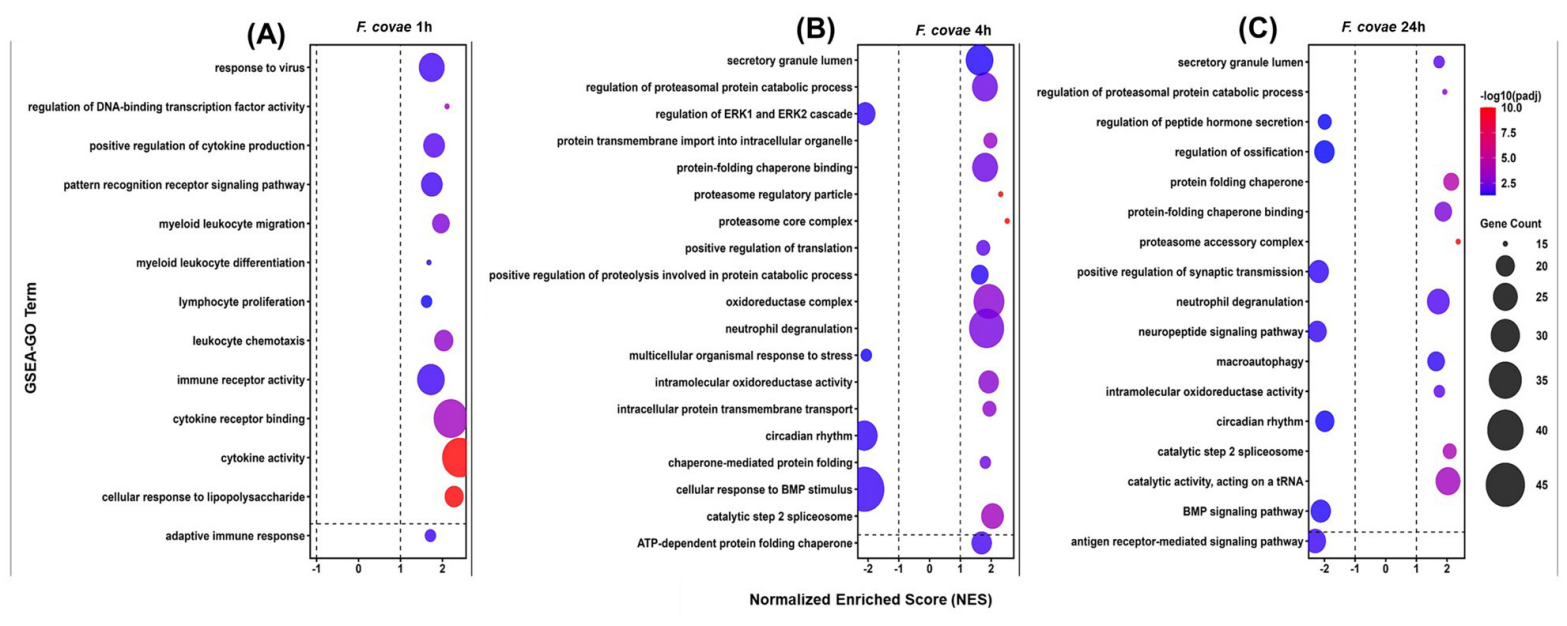
Gene Set Enrichment Analysis (GSEA) showing functional groups significantly enriched in response to *F. covae* infection. **(A)** 1 h post-infection, **(B)** 4 h post-infection, and **(C)** 24 h post-infection. The x-axis represents the Normalized Enrichment Score (NES), indicating the level of gene set enrichment. The y-axis lists enriched Gene Ontology (GO) terms. The size of the dots represents the number of genes associated with each GO term and the color represents statistical significance. The dotted vertical lines mark the significance threshold.

To investigate the dynamic biological responses to *F. covae* infection, we performed Gene Set Enrichment Analysis (GSEA) on ranked gene lists at three post-infection time points: 1, 4, and 24 h. The enrichment of Gene Ontology (GO) terms related to biological processes (BP), molecular functions (MF), and cellular components (CC) was analyzed to identify key pathways activated during infection (Figure 5 and Supplementary Table 5).

At 1 h post-infection, several immune-related GO terms were significantly enriched (Figure 5A and Supplementary Table 5). These included response to viruses, cytokine activity, immune receptor activity, leukocyte chemotaxis, and pattern recognition receptor signaling pathways. The enrichment of cytokine receptor binding (GO:0005126) and positive regulation of cytokine production (GO:0001819) suggests the activation of cytokine-mediated immune signaling. The cellular response to lipopolysaccharide (GO:0071222) indicates a rapid recognition of bacterial components, leading to an early immune response. Additionally, myeloid leukocyte migration (GO:0097529) and adaptive immune response (GO:0002250) highlight the recruitment of immune cells to the site of infection.

By 4 h post-infection, the enriched GO terms shifted toward pathways associated with protein degradation, oxidative stress responses, and immune cell function (Figure 5B and Supplementary Table 5). Notably, regulation of the proteasomal protein catabolic process (GO:0061136) and proteasome core complex (GO:0005839) were significantly enriched, suggesting an upregulation of proteasomal degradation, likely involved in antigen processing and cellular stress response. The regulation of ERK1 and ERK2 cascade (GO:0070372) indicates involvement of mitogen-activated protein kinase (MAPK) signaling, which plays a role in inflammatory responses. Furthermore, neutrophil degranulation (GO:0043312) suggests the activation of innate immune cells to combat bacterial invasion. The enrichment of oxidoreductase complex (GO:1990204) and intramolecular oxidoreductase activity (GO:0016860) indicates oxidative stress response, which could contribute to the host defense mechanism or bacterial pathogenesis.

At 24 h post-infection, there was a notable shift toward pathways involved in protein homeostasis, cellular adaptation, and recovery mechanisms (Figure 5C and Supplementary Table 5). The upregulation of protein-folding chaperone binding (GO:0051087) and the proteasome accessory complex (GO:0022624) suggests a persistent cellular stress response requiring chaperone-mediated stabilization and enhanced proteasomal turnover. Strong enrichment of ribosome biogenesis and mitochondrial protein-targeting pathways further indicates increased biosynthetic and metabolic activity as cells attempt to restore homeostasis. In contrast, several neuroendocrine-associated pathways, including neuropeptide signaling (GO:0007218), regulation of peptide hormone secretion (GO:0090276), and circadian rhythm (GO:0007623), were significantly negatively enriched, reflecting suppression of neuropeptide-mediated communication and circadian regulatory processes during the later phase of infection. Additionally, enrichment of macroautophagy (GO:0016236) supports a transition toward autophagic clearance of damaged proteins or cellular debris as part of ongoing cellular adaptation.

#### 3.3.2 Gene ontology following acute CuSO_4_ exposure and immediate *F. covae* challenge

To address the functional profile of the acute CuSO_4_ plus immediate *F. covae* challenge group, GSEA on ranked gene lists was conducted on this group. Since no DEGs were detected at 1 h post-challenge, enrichment analysis did not yield a 1 h GO profile. At 4 h post-challenge, immediate CuSO_4_ exposure produced a transcriptomic profile dominated by cellular stress responses and minimal immune activation. While only 45 DEGs were detected, GSEA GO terms showed strong positive enrichment of mitochondrial and proteostasis-related pathways, including mitochondrial large ribosomal subunit (GO:0005762), mitochondrial translational elongation (GO:0070125), and protein folding (GO:0006457), reflecting copper-induced oxidative and proteotoxic stress. Concurrently, negatively enriched pathways encompass microfilament motor activity (GO:0000146), myosin II complex (GO:0016460), and sarcomere organization (GO:0045214), possibly indicating suppressed cytoskeletal integrity and reduced contractile function within gill epithelia. Notably, PRR- and cytokine-associated genes remained largely unresponsive even by 4 h, suggesting that the gill prioritized stress mitigation and structural stabilization over immune activation in the immediate hours following *F. covae* exposure.

At 24 h post-challenge, gill transcriptomes displayed a strong shift toward genome maintenance, oxidative stress management, and vesicular transport, with several high-confidence positively enriched pathways, including nuclear DNA replication (GO:0033260), DNA replication initiation (GO:0006270), replication fork (GO:0005657), double-strand break repair via homologous recombination (GO:0000724), proton-transporting ATPase complex (GO:0016469), ER-to-Golgi vesicle-mediated transport (GO:0006888), and cellular response to reactive oxygen species (GO:0034614). Notably, several innate immune pathways also became positively enriched at this time point, including neutrophil degranulation (GO:0043312), azurophil granule lumen (GO:0035578), and ficolin-1-rich granule lumen (GO:1904813), indicating that innate effector functions that were largely absent at 4 h begin to re-engage by 24 h, suggesting partial restoration of immune responsiveness following acute copper-induced suppression. In contrast, negatively enriched categories remained centered on epithelial structure and contractility, including collagen trimer (GO:0005581), extracellular matrix structural constituent conferring tensile strength (GO:0030020), myosin II complex (GO:0016460), and myosin filament (GO:0032982), reflecting persistent ECM remodeling and reduced contractile integrity.

#### 3.3.3 Gene ontology CuSO_4_ exposure with 24 h recovery followed by *F. covae* challenge

**Figure 6:**
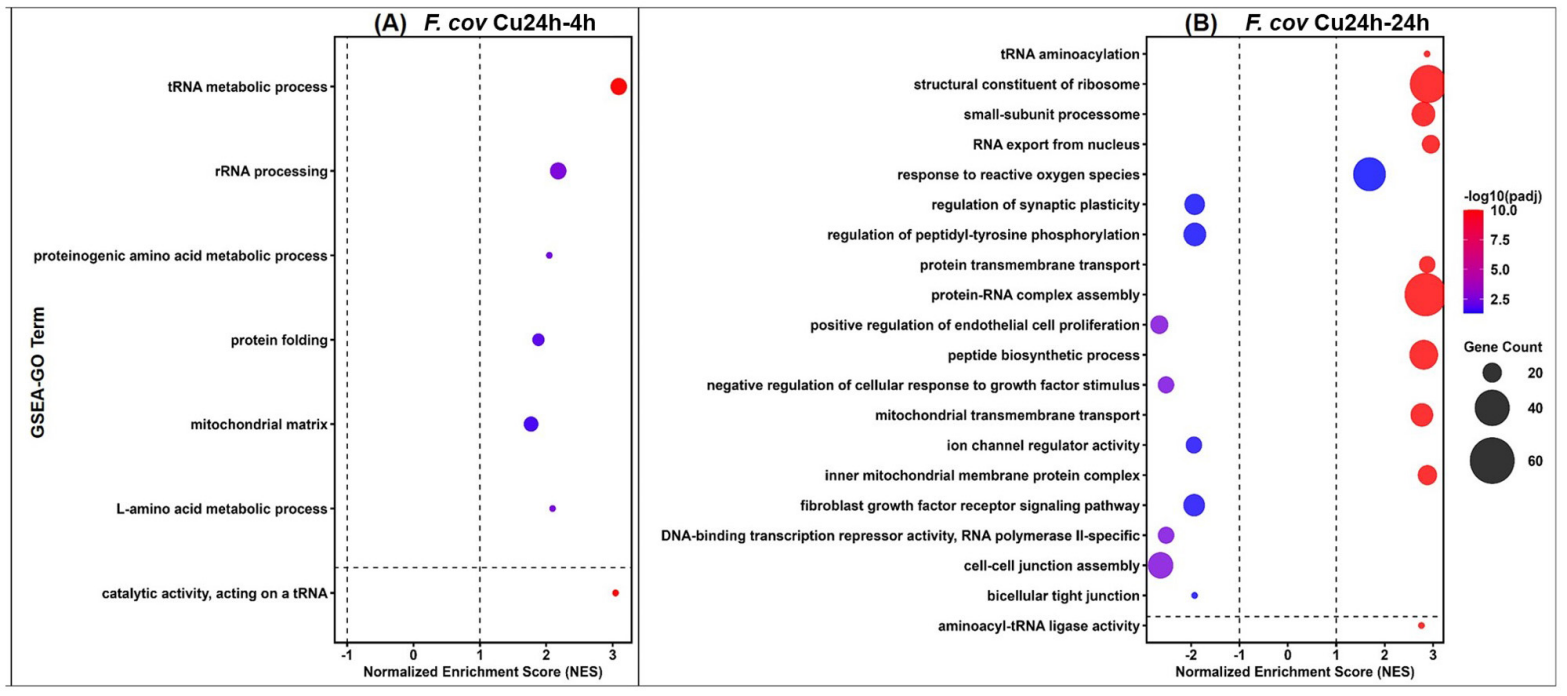
Gene Set Enrichment Analysis (GSEA) showing functional groups significantly enriched in response to *F. covae* infection after treatment with copper sulfate and allowing for 24 h recovery period. **(A)** 4 h post-infection, **(B)** 24 h post-infection. The x-axis represents the Normalized Enrichment Score (NES), indicating the level of gene set enrichment. The y-axis lists enriched Gene Ontology (GO) terms. The size of the dots represents the number of genes associated with each GO term and the color represents statistical significance. The dotted vertical lines mark the significance threshold.

To determine the molecular response to infection after CuSO_4_ exposure, GSEA on ranked gene lists at 4 h and 24 h post-exposure was examined, as no significantly enriched GO terms were identified at 1 h. The enrichment of Gene Ontology (GO) terms related to biological processes (BP), molecular functions (MF), and cellular components (CC) was analyzed to determine pathways activated under copper-induced stress (Figure 6 and Supplementary Table 5). At 4 h post-exposure, a limited number of GO terms including pathways related to RNA metabolism and protein processing were significantly enriched (Figure 6A and Supplementary Table 5). The most highly enriched GO terms included tRNA metabolic process (GO:0006399), rRNA processing (GO:0006364), and proteinogenic amino acid metabolic process (GO:0170039). Catalytic activity, acting on a tRNA (GO:0140101), was also enriched, indicating enhanced tRNA modification and translation-related processes, likely in response to cellular stress. The strong enrichment of protein folding and mitochondrial matrix suggests that infection with *F. covae* after treatment with CuSO_4_ and allowing for a recovery period induces early mitochondrial gene involvement and protein homeostasis pathways.

By 24 h post-exposure, the biological processes activated in response to CuSO_4_ exposure were markedly different (Figure 6B and Supplementary Table 5). Notably, oxidative stress-related pathways were significantly enriched, with response to reactive oxygen species (GO:0000302) and protein targeting to mitochondrion (GO:0006626) being over-expressed. Additionally, enriched terms such as regulation of peptidyl-tyrosine phosphorylation (GO:0050730), protein transmembrane transport (GO:0071806), and positive regulation of endothelial cell proliferation (GO:0001938) suggest cellular adaptation and repair mechanisms were negatively enriched due to this prolonged metal exposure. Although this group mounted a more effective overall response to *F. covae*, the lingering structural effects of CuSO_4_ exposure were evident from the downregulation of cell–cell junction assembly (GO:0007043) and bicellular tight junction (GO:0005923), indicating that epithelial barrier repair processes were still suppressed despite improved immune function. Likewise, the downregulated enrichment of the fibroblast growth factor receptor signaling pathway (GO:0008543) suggests that growth-factor-mediated tissue remodeling was not fully engaged at 24 h post-infection.

### 3.4 Immune and stress associated gene expression patterns

**Figure 7:**
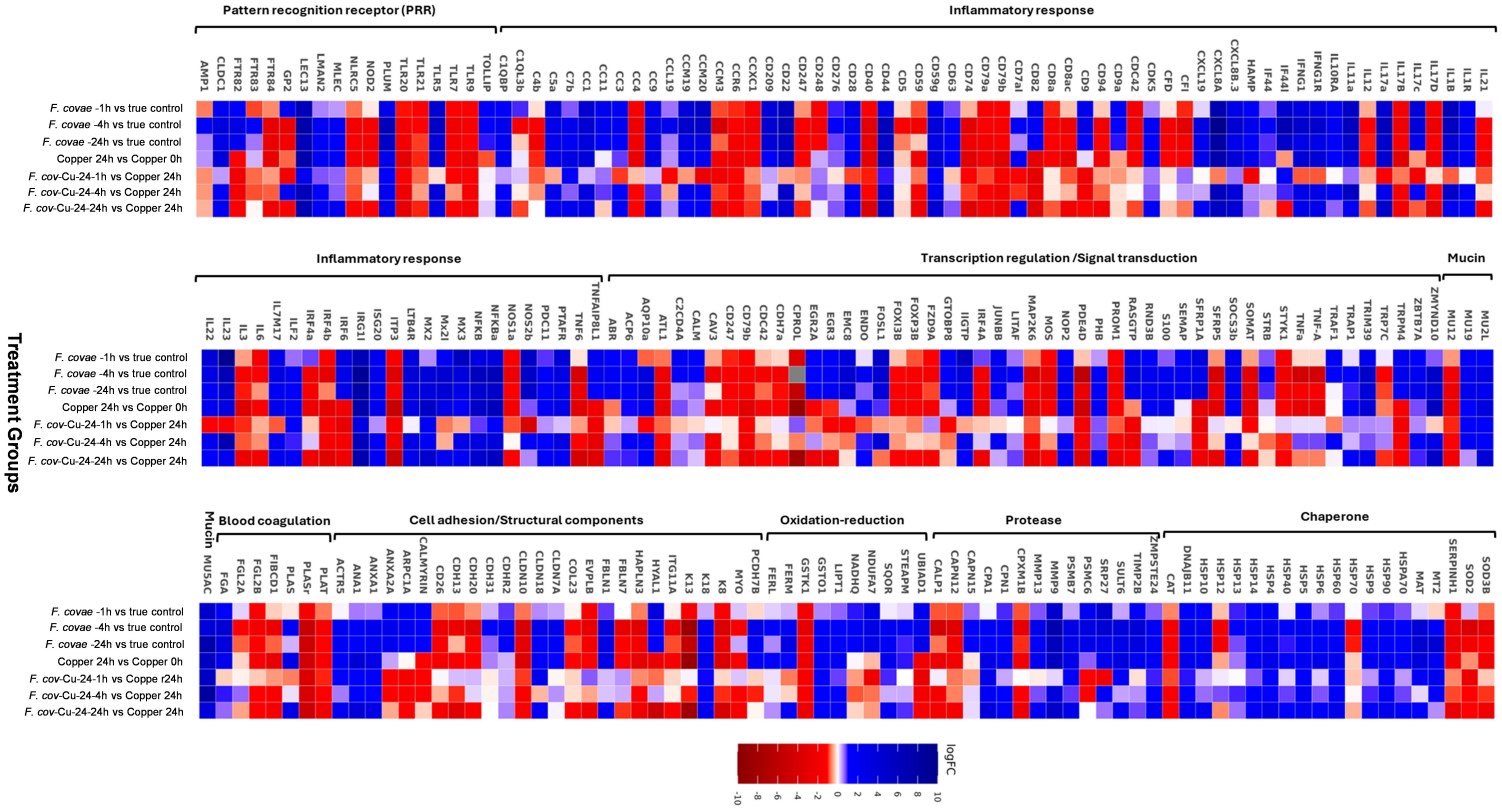
Heatmap showing select immune-related genes in response to *F. covae* with or without copper sulfate treatment. Pairwise comparisons shown include *F. covae* infection compared to control over time and *F. covae* infection after copper sulfate recovery group over time.

Across infection conditions, 234 immune and stress-related genes showed significant differential expression (Supplementary Table 6; Figure 7). In *F. covae*-only infected fish (no copper treatment), early time-points were characterized by upregulation of core PRRs (including TLR5), cytokines (IL-23, IL-17), chemokines (CXCL8a), and interferon-related genes (IFNG1R, IFN-γ). Adaptive immune markers (CD22, CD44) became strongly upregulated over the course of infection. In contrast, acute CuSO_4_ exposure suppressed multiple PRRs (NOD2, NLRC5), while metallothioneins, heat-shock proteins, and oxidative-stress genes were significantly perturbed, consistent with acute metal stress. Recovery for 24 h restored immune responsiveness, enabling robust PRR activation, chemokine production, neutrophil-related pathways, and antigen-presentation processes following infection.

### 3.5 qPCR validation

RT-qPCR analysis of five genes (IL-1β, MMP9, APRT, Blec, IRG1) showed expression patterns consistent with RNA-seq results across treatments and timepoints (Supplementary Table 7), confirming the reliability of the RNA-seq data.

## 4 Discussion

Copper sulfate is used to manage disease conditions in freshwater fishes, including skin lesions and parasite control (11, 16, 17, 21, 22, 35). Previous warmwater fish studies have explored the effect of CuSO_4_ on various bacteria (14, 48). Notably, Farmer et al. (7, 14) reported a significantly higher survival rate of channel catfish challenged with *F. columnare* when they were exposed to CuSO_4_ and allowed a 24 h recovery period compared with those that were not allowed to recover. However, the broader impact of CuSO_4_ on the channel catfish immune system function has not been fully explored. This study utilized bulk RNA sequencing to understand global gene expression changes due to Cu stress as well as innate and adaptive immune gene expression differences among channel catfish challenged with *F. covae* at specific CuSO_4_ conditions, which may be beneficial in the improvement of management strategies and the development of therapeutants for use in aquaculture farms.

### 4.1 Differential gene expression over time-course of infection with *F. covae*

Pattern recognition receptors (PRRs) are germline-encoded receptors that recognize pathogens, and toll-like receptors (TLRs) are PRRs that recognize microbial components (49). At 1 h post-challenge, PRRs (GO:0002221) were significantly upregulated. The significant upregulation of TLR5 highlights the recognition of a component of *F. covae*. In channel catfish, two forms of TLR5 have been reported (50) and although these receptors classically detect flagellin in mammals (51), the strong induction of TLR5 here suggests signaling might be activated by non-canonical bacterial pathogen-associated molecular patterns (PAMPs), possibly mediated or amplified through coordination with other PRRs. Additionally, pro-inflammatory cytokines, including interleukin-23 (IL-23) and interleukin-1β (IL-1β) were significantly upregulated suggesting an early activation of inflammatory pathways. In mammals, IL-23 production sustains the differentiation of Th17 cells, which serve as the main cellular source of other pro-inflammatory cytokines, and IL-1β also serves as a primary mediator for early pro-inflammatory activities (52–54). These cytokines are crucial in recruiting other immune cells and initiating pathogen clearance.

At 4 h, the immune response shifted toward neutrophil degranulation (GO:0043312). Neutrophils are critical components of teleost immunity and play several roles in extracellular and intracellular antimicrobial defenses (55). In teleost, the CXC chemokine CXCL8 is responsible for cell migration and bacterial invasion (56). The upregulation of CXCL8a suggests the recruitment and migration of neutrophils, ensuring that they release antimicrobial substances to fight off the pathogen. In addition to neutrophil recruitment, the expression of the pro-inflammatory cytokines IL-23 and IL-17a/f1 were sustained. The upregulation of IL-17a/f1 suggests the involvement of the Th17 immune response, which is critical for promoting inflammation and enhancing the neutrophil response (53). Moreover, the continued upregulation of TLR5 at 4 h reinforces its dominant role in pathogen recognition and immune activation during the early stages of infection.

At 24 h, the pathway that was significantly upregulated was antigen receptor-mediated signaling (GO:0050851). Immune cell markers were significantly upregulated, including CD22 and CD44a, suggesting the involvement of B and T cells, which play crucial roles in the adaptive immune system. These immune cell markers have also been found in the ocular mucosa of rainbow trout at the late stages of *F. columnare* infection (57). In mammals, CD22 is a down regulator of antigen receptor signaling and is central to peripheral B cell homeostasis (58, 59). CD44 is an adhesion molecule crucial for mediating T-cell extravasation, responses, and development (60–62). The upregulation of these two receptor molecules suggests both the regulation of B-cell activation and antibody production and the migration of T-cells to fight *F. covae* infection. CXCL8a was still upregulated as well as TLR5, suggesting that even though the immune response at 24 h is shifting toward adaptive immunity, the innate immune system response is sustained. Inflammatory genes play a vital role in regulating the immune response during infection or injury. These genes encode proteins that help to control inflammation, a process that protects the fish by removing harmful stimuli, such as pathogens or damaged cells, and begins the healing process. C5a (Complement Component 5a Receptor 1) is a G-protein-coupled receptor involved in the activation of the complement system. C5a is a potent inflammatory mediator that recruits immune cells like neutrophils and macrophages to the infection site. It also promotes the release of pro-inflammatory cytokines, intensifying the inflammatory response (63, 64). CXCL9 (C-X-C Motif Chemokine Ligand 9) is a chemokine that recruits T-cells, natural killer (NK) cells, and other immune cells to the site of infection. It is induced by interferon-gamma (IFN-γ) and plays a critical role in immune surveillance and inflammation (65, 66). CXCL8a (C-X-C Motif Chemokine Ligand 8a, also known as IL-8) is a key chemokine involved in the recruitment of neutrophils to infection sites. It also plays a role in promoting angiogenesis during tissue repair and at initial upregulation. It helps in the recruitment of dendritic cells and T-cells to lymphoid tissues, enabling the activation of adaptive immune responses (66, 67). Furthermore, the moderate increase in expression across all time points shows that CCL19 may be contributing to the coordination between the innate and adaptive immune responses during infection (16, 68, 69).

Overall, the temporal gene expression data demonstrate that when channel catfish is exposed to *F. covae*, there is an initial innate immune response with a gradual transition into adaptive immunity. However, the inflammatory mechanisms triggered by the innate immune system continue even through the later stages of infection.

### 4.2 Differential gene expression in catfish exposed to CuSO_4_ and *F. covae* simultaneously

The transcriptional responses of channel catfish gills to simultaneous CuSO_4_ exposure and *F. covae* challenge reveal a markedly different pattern than *F. covae* challenge alone. Rather than mounting moderate or escalating immune activation, the gill exhibited a profoundly suppressed early immune response at both 1 h and 4 h post-challenge, consistent with copper's ability to induce oxidative and proteotoxic stress in fish tissues (16, 17, 19, 70–72). At 1 h, no significant DEGs were detected, indicating that CuSO_4_ exposure effectively blunted the earliest transcriptional responses to infection. By 4 h, this suppression became more apparent. Gene expression was strongly enriched for mitochondrial translation (GO:0070125), protein folding (GO:0006457), unfolded protein binding (GO:0051082), and ATP-synthase-related processes (GO:0016469), reflecting a cellular state dedicated to stabilizing damaged proteins, restoring mitochondrial function, and mitigating copper-induced injury (16, 17, 19, 70–72). During this same window, immune pathways remained notably absent, and structural components including cytoskeletal motor proteins, myosin filaments, and actin-based contractile elements were prominently downregulated, suggesting that epithelial integrity and innate immune competency were both compromised with acute CuSO_4_ exposure (26–29).

By 24 h post-challenge, however, the copper-suppressed transcriptional landscape began to shift. Although extracellular matrix organization and contractile elements remained downregulated, the dominant features of the transcriptome included robust activation of DNA replication, replication-fork extension, homologous recombination repair, ER-Golgi vesicle transport, and cellular antioxidant responses, indicating widespread cellular recovery and metabolic reorganization (16, 17, 19, 71, 72). Several innate immune pathways also became positively enriched at this timepoint, most notably neutrophil degranulation (GO:0043312) and multiple granule-associated effector processes, hallmarks of activated mucosal immunity in teleosts (55–57). These findings indicate that a partial restoration of immune responsiveness emerges only by 4–24 h, once the initial copper-induced stress burden has begun to resolve. Fish in this group enter infection with gills seemingly overwhelmed by copper-induced stress responses and, therefore, lack the capacity to detect and respond effectively to *F. covae* during the early hours of infection, explaining their markedly heightened susceptibility (7, 8, 14, 23, 24). Overall, our gene expression results indicate that when channel catfish are simultaneously exposed to CuSO_4_ and *F. covae*, the copper-induced oxidative and proteotoxic stress suppresses key pattern-recognition receptors, including NOD2 and NLRC5, consistent with previous findings (70, 72).

### 4.3 Differential gene expression in catfish treated with CuSO_4_ and exposed to *F. covae* after a 24 h recovery period

The genes expressed in channel catfish treated with CuSO_4_ and challenged with *F. covae* after a 24 h recovery period were investigated in this group. In gill tissues sampled after 1 h post-infection, there was significant downregulation of key PRRs, including NLRC5, TLR7, NOD2, and TLR21, indicating an attenuated early PRR-mediated response. Unlike in the other treatment group, where TLR5 was consistently upregulated, TLR5 was downregulated but not significantly. Despite this early suppression of PRRs, the pathway associated with cellular response to lipopolysaccharide (GO:0071222) was activated, suggesting that some innate sensing mechanisms were still responsive to bacterial PAMPs. The observed PRR downregulation likely reflects residual copper-induced stress (16, 22, 68), which may transiently limit catfish ability to mount an effective early immune response. However, interferon signaling was strongly induced, as indicated by the upregulation of IFI44L. Additionally, CD209 antigen-like protein E, involved in pathogen recognition and antigen presentation, was upregulated, indicating that innate surveillance was not completely suppressed. The significant induction of irg1l, a gene associated with phagosome activity and apoptosis, further supports the presence of a partially active antimicrobial response despite reduced early PRR signaling (73).

At 4 h post-infection, channel catfish that received a 24 h recovery period after CuSO_4_ exposure mounted a much stronger and more coordinated immune response than was observed at 1 h. TLR5 was highly upregulated, reflecting the recovery of PRR-mediated pathogen sensing of *F. covae*. The neutrophil degranulation pathway (GO:0043312) was significantly enriched, supported by a strong induction of CXCL8a, which promotes neutrophil recruitment during bacterial infection (56). Compared to 1 h, expression of irg1l continued to increase, reflecting activation of antimicrobial metabolic pathways and regulation of inflammation and tissue repair (53, 54, 74). Moreover, the strong upregulation of IL-23 suggests that a coordinated inflammatory response was being maintained, likely through the activation of Th17-asssocated signaling (52), which is consistent with sustained neutrophil activation (53, 54). Adaptive immune markers, including CD44, CD22, and IL-23, were also strongly upregulated, indicating early engagement of lymphocyte-mediated processes (60–62). Together, these results show that after sufficient recovery from copper-induced stress, channel catfish can mount a rapid and coordinated innate and adaptive immune response to *F. covae* by 4 h post-challenge.

At 24 h post-infection, channel catfish that received a 24 h recovery period after CuSO_4_ exposure exhibited the strongest and most comprehensive transcriptional response among the recovery groups. A total of 3,925 DEGs were detected, with 1,553 genes upregulated and 2,372 downregulated, indicating widescale activation of both innate and adaptive immune processes. Antigen processing and presentation (GO:0002478) pathways were significantly enriched, reflecting a transition toward a more complex and targeted immune response. The continued upregulation of CD209 antigen-like protein E, a lectin receptor involved in pathogen recognition and antigen delivery to T-cells (75), highlights activation of antigen-presenting cell functions. Additionally, the induction of complement component C7b, a major effector in clearing bacteria through the Membrane Attack Complex (MAC) (63, 76), together with enrichment of the antimicrobial humoral response (GO:0019730), indicates strong complement-mediated immune activity by 24 h. Pro-inflammatory cytokines such as IL-17a/f1 and the chemokine CXCL8a also remained significantly elevated, suggesting sustained neutrophil recruitment and ongoing resolution of infection-related tissue damage. Taken together, these findings indicate that, by 24 h post-challenge, catfish that recovered from copper treatment mount a fully integrated and robust immune response, engaging antigen presentation, complement activation, lymphocyte-mediated processes, and sustained innate effector pathways to combat *F. covae*.

### 4.4 Broader aquaculture and one health implications

Beyond the direct immunological effects observed in channel catfish, these findings have broader implications for aquaculture resilience and One Health outcomes. Antimicrobial use in aquaculture is increasingly recognized as a contributor to antimicrobial resistance (AMR), with resistant bacteria capable of moving among aquatic environments, farmed animals, and humans (77, 78). Disease-management strategies that reduce antibiotic dependency, such as optimizing CuSO_4_ dosing combined with exposure/recovery timing, may help mitigate selective pressures that drive AMR emergence (79). Environmental considerations are similarly important because copper can accumulate in sediments, impact non-target organisms, and alter microbial communities (80). Refinements to CuSO_4_ application protocols that maintain fish health while minimizing ecological loading support safer aquatic production systems and align with One Health principles linking environmental quality, animal health, and food safety (81, 82).

## 5 Conclusions

This study demonstrates that CuSO_4_ exposure has a time-dependent impact on the immune competence of channel catfish during *F. covae* infection. Acute exposure suppresses early pattern-recognition receptors such as TLR5, NOD2, and NLRC5, delaying immune activation and increasing disease susceptibility. In contrast, a 24 h recovery period restores immune readiness, enabling coordinated innate and adaptive responses and significantly improving survival to *F. covae*. These findings highlight the importance of treatment timing when using copper sulfate in aquaculture systems. Optimizing CuSO_4_ protocols to include adequate recovery periods may help balance pathogen control with preserved immune function. Future work should examine the longer-term physiological impacts of copper exposure and evaluate strategies that mitigate oxidative stress while maintaining disease management efficacy.

## Author's note

All opinions expressed in this paper are the author's and do not necessarily reflect the policies and views of USDA, DOE, or ORAU/ORISE. Mention of trade names or commercial products in this publication is solely for the purpose of providing specific information and does not imply recommendation or endorsement by the United States Department of Agriculture. The USDA is an equal opportunity provider and employer.

## Data Availability

The datasets presented in this study can be found in online repositories. The names of the repository/repositories and accession number(s) can be found below: https://www.ncbi.nlm.nih.gov/geo/, GSE136222.

## Supplementary material

Supplementary Table 1: Total differentially expressed genes (DEGs) from pairwise comparison of channel catfish gill tissue samples collected at 1, 4, and 24 h post-infection with *F. covae*. Data includes gene accession numbers, log2 fold-change values, the false discovery rate (FDR < 0.05), and the gene description provided from the annotated channel catfish genome assembly.

Supplementary Table 2: Total differentially expressed genes (DEGs) from pairwise comparisons of channel catfish gill tissue samples collected after fish were exposed to 2.1 mg/L copper sulfate and then immediately challenged with *F. covae*. Samples were collected at 1, 4, and 24 h post-infection. Data includes gene accession number, log2 fold-change values, the false discovery rate (FDR < 0.05), and the gene description provided from the annotated channel catfish genome assembly.

Supplementary Table 3: Total differentially expressed genes (DEGs) from pairwise comparison between channel catfish gill tissue samples collected after fish were exposed to 2.1 mg/L copper sulfate and allowed a 24 h recovery period and those exposed to copper sulfate and then sampled. Data includes gene accession number, log2 fold-change values, the false discovery rate (FDR < 0.05), and the gene description provided from the annotated channel catfish genome assembly.

Supplementary Table 4: Total differentially expressed genes (DEGs) from pairwise comparisons of channel catfish gill tissue samples collected after fish were exposed to 2.1 mg/L copper sulfate and allowed a 24 h recovery period followed by challenge with *F. covae*. Samples were collected at 1, 4, and 24 h post-infection. Data includes gene accession number, log2 fold-change values, the false discovery rate (FDR < 0.05), and the gene description provided from the annotated channel catfish genome assembly.

Supplementary Table 5: An Excel sheet with Gene Ontology (GO) terms analyzed by Gene Set Enrichment Analysis (GSEA) for each pairwise comparison, where significant (FDR < 0.05).

Supplementary Table 6: Differential expression of immune-relevant genes in the gills of channel catfish following copper sulfate treatments and followed by challenged with virulent *F. covae*. The significant fold change at false discovery rate (FDR) is bold. Numbers of fold change are expressed in log2 value.

Supplementary Table 7: Reverse-transcription quantitative PCR (RT-qPCR) of select genes compared to RNA-seq values. Fold-changes represent the ratio of gene expression at a given timepoint relative to control and normalized to the housekeeping gene.
